# Robust transport of high-speed data in a topological valley Hall insulator

**DOI:** 10.1515/nanoph-2025-0298

**Published:** 2025-11-13

**Authors:** Byoung-Uk Sohn, George F. R. Chen, Hongwei Gao, Doris K. T. Ng, Dawn T. H. Tan

**Affiliations:** Photonics Devices and System Group, 233793 Singapore University of Technology and Design, Singapore 487372, Singapore; Institute of Microelectronics, Agency for Science Technology and Research (A*STAR), 2 Fusionopolis Way, #08-02, Innovis Tower, Singapore 138634, Singapore

**Keywords:** topological photonics, valley Hall insulator, Kagome lattice, pulse amplitude modulation 4-level high-speed data transmission

## Abstract

Photonic topological insulators provide robust transport of light, enabling interesting phenomena such as unidirectional light propagation and immunity to disorder. The discovery of how to effectively break time reversal symmetry was an important development in the field of photonic topological insulators. Knowledge on how to implement designs in all-dielectric systems was an especially crucial development, enabling complementary metal-oxide semiconductor-based materials and processes to be used to study such structures, accelerating their pace of innovation. On the other hand, transmission of high-speed data is of fundamental importance in communications systems prolific in data centers and telecommunications. In this paper, we demonstrate robust transport of high-speed non-return-to-zero (NRZ) and pulse amplitude modulation 4 (PAM4) in a photonic topological insulator based on the quantum valley Hall effect. The structure utilizes a Kagome lattice with a slightly broken symmetry to achieve a domain wall between two regions with half-integer valley Chern numbers. The topological structure’s immunity to backscattering allows high-speed data to be transmission through a zigzag path with four 120° bends. Characterization of reference devices including a trivial device and photonic waveguide device shows that the topological device is superior in the robust transport of high-speed data, enabling a low BER of 10^−8^ for 30 Gbps NRZ data and an open eye observed for 100 Gbps PAM4 data even when transmitted through a zigzag optical path.

## 1 Introduction

The ability for photonic devices to transmit data at high speeds is more important than ever before given the boom in telecommunications and artificial intelligence, the latter of which requires extensive computational power at high speeds at low energy consumption [1]. Topology offers protection against certain types of defects in the transmission of light and offers a promising pathway towards robust light propagation and implementation of optical phenomena [2], [3], [4], [5], [6], [7], [8], [9], [10], [11], [12], [13], [14], [15]. In particular, valley Hall photonic crystals are an interesting class of photonic devices which allow topological insulator properties to be implemented, enabling topologically protected boundary states immune to defects to form, as long as chiral symmetry is preserved. A propagation boundary between two topological domains exists when the topological invariant, the Chern number, differs in both. Secondly, the valley Hall state uses spatial symmetry in place of using an internal degree of freedom such as spin (polarization). Consequently, the topological state can be easily implemented in photonics devices. The valley Hall insulator has been reported in several domains, including acoustic valley Hall insulators and plasmonic valley Hall insulators. These valley Hall insulators were previously demonstrated by leveraging the topological behavior 2D MoS_2_ monolayers [16], [17] and bilayer graphene [18], [19], [20], [21], [22]. The valley points K and K′ in momentum space of a crystal have a role of spin in the spin Hall effect. The K and K′ valley points are singular points in momentum space. As a result of the topological singularity, the global phase of the E-field has a closed path at the K and K′ points, generating a Berry phase of ∓*π*. The boundary between two bulk states, K and K′, give rise to a robust, topologically protected pathway. At this interface, robust light transport is observed even in the presence of sharp edges, although this robustness is not perfectly immune to back-scattering, as previously reported [23], [24]. Conversely, a non-topologically protected photonic crystal would experience significant optical losses from these abrupt interfaces [25]. A number of applications harnessing this robust transmission of light even in the presence of intentionally or unintentionally imposed defects have been reported, including directional couplers and light transport [26], [27], [28], [29], [30], lasing [31], slow light [32], switches [33], [34], [35], [36], wavelength converters [25] and valley-locked waveguide transport in acoustic heterostructures [37].

In this work, we demonstrate high-speed data transmission through a topological valley Hall photonic device robust against backscattering. Superior high-speed transmission properties are observed compared to non-topological photonic devices and conventional photonic waveguides. We demonstrate 50 Gbps non-return-to-zero (NRZ) and 100 Gbps pulse amplitude modulation 4 (PAM4) high-speed data transmission guided through topologically protected valley Hall boundary states, robust against backscattering when propagated through a boundary with four 120 °C bends. The performance of high-speed data transmission is compared between the topological photonic device, non-topological photonic crystal and a photonic waveguide, and is shown to enable significantly better bit error rates and eye diagrams, indicating robust high-speed transport of optical data. The zig zag configure is the shape of propagation to implement a challenging propagation path for high-speed data, so as to explore topological protection conferred by the valley Hall photonic crystal. In regular waveguides or non-topological waveguides, backscattering happens when light encounters sharp edges owing to sudden change of propagation direction. The slightly changed direction has stiffness in the point and a changed effective index. The changed effective index can induce backscattering. For topological devices, the boundary state is robust against backscattering by the locally distorted effective refractive index because the topological behavior is a long range of property related to the Berry phase. The non-topological device and topological device is implemented in slightly narrow bands. Thus, the defect state possesses a high group index. The backscattering is the dominant loss mechanism in the region because linear loss is *α* = *An*_*g*_ + *Bn*_*g*_^2^, where *An*_*g*_ is out-of-plan scattering and *Bn*_*g*_^2^ is backscattering term. Back scattering in the topological device is low although the propagation through a zigzag path is typically a challenging propagation path which leads to significant scattering loss, can also be seen from the results we report for the zigzag photonic waveguide and zigzag non-topological photonic crystal in this paper.

## 2 Topological valley Hall photonic crystal design

**Figure 1:**
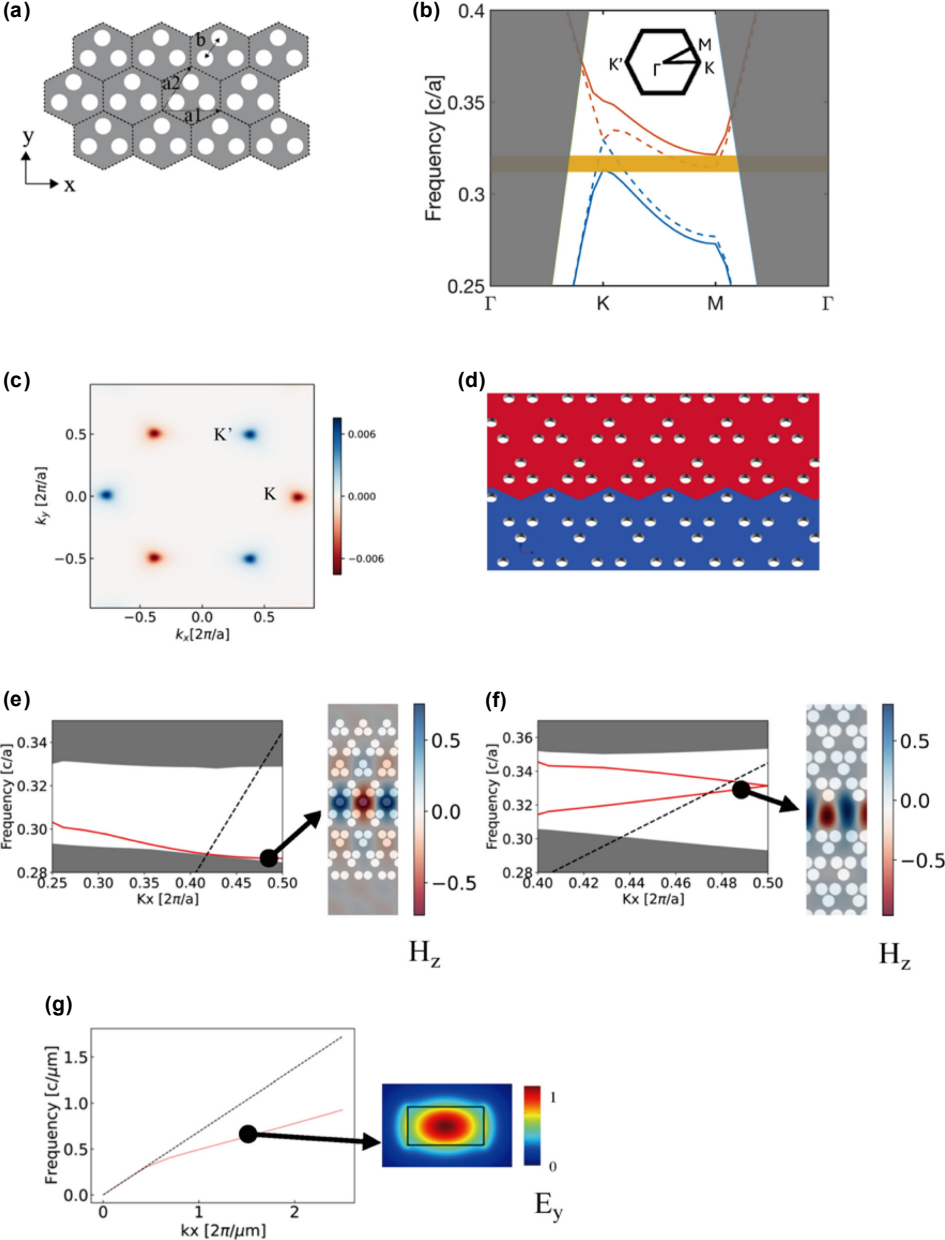
The topological structure, bands, and modes for the photonic devices. (a) The photonic crystal with Kagome symmetry. (b) The TE band energy band diagram for the Kagome symmetry (dashed lines) slightly broken Kagome symmetry, *b* = 1.1*b*_0_ (bold lines). (c) The Berry curvature for the slightly broke Kagome symmetry. (d) The topological insulator with topological boundary. (e) The band diagram and TE mode shape for the topological photonic insulator. (f) The band diagram and TE mode shape for the trivial defect photonic crystal. (g) The band diagram and TE mode shape for the conventional waveguide.

Our device utilizes Kagome symmetry which has Honeycomb unit cell with a three-hole basis, with unit vectors a1 and a2. The distance between each hole within the basis is $b=b_{0}=\frac{a}{2\sqrt{3}}$ , where *a* is the lattice constant. In this work, *a* has a value of 0.49 μm. The contact points of bands, K and K′, are singular points, which give us nontrivial topology. When the Kagome symmetry is slightly broken such that *b* = 1.1*b*_0_, the contact point of bands for the Kagome crystal (dashed lines in Figure 1(b)) is widened, resulting in the forbidden band (yellow region). The irreducible Brillouin zone has symmetry points in the crystalline wavevector region, Γ, *K*, *M*. K, K′ are singular points in momentum space. They have non-zero berry curvature even though the symmetry is slightly broken as shown in Figure 1(c). The Berry curvature can be calculated using $\Omega \left(k\right)=\nabla k\times A\left(k\right)$ , where *A*(*k*) represents the Berry connection, which is defined as $A\left(k\right)=i\left\langle u\left(k\right)\left|\nabla k\right|u\left(k\right)\right\rangle$ , where $\left|u\left(k\right)\right\rangle$ denotes the normalized Bloch wave function obtained through numerical simulations. The local calculation of valley Chern numbers near the K(K′) valley for the lower band, based on a surface integral of the Berry curvature, yields *C*_K_ = −1/2 and *C*_K_′ = 1/2. As *b* = 0.9*b*_0_, the photonic crystal undergoes a topological transition, leading to a sign reversal in the associated valley Chern numbers. Consequently, at the domain wall between two valley surface-wave photonic crystals with opposite half-integer valley Chern numbers, the valley-protected topological charge difference across the interface is quantized (∣*C*_K_−*C*_K_′∣ = 1), confirming the presence of a single chiral edge state per valley propagating along the interface. The generated boundary state is a spatially localized state in the forbidden band gap from the slightly broken symmetry. The band structure is calculated as shown in Figure 1(e), the grey region represents bulk states, and red line is the generated topological boundary state, whereas the dashed line indicates the light cone in the cladding material. The mode shape is well localized on the boundary between two different bulk topologies.

We further prepare two photonic devices for reference experiments. These include the trivial defect photonic device and a conventional photonic waveguide. For a fair comparison between the three types of waveguides, we adopt the same 120° sharp bends for the zigzag path for all three systems. For the trivial defect photonic device, we design a single defect line in the slightly broken Kagome crystal – Two defect modes are generated as shown as the red lines in the Figure 1(f). The mode shape is well localized within the defect in the photonic crystal. The conventional waveguide utilizes a USRN core and SiO_2_ cladding. The waveguide has width and height of 600 nm and 300 nm, respectively. The corresponding band diagram for the waveguide’s TE mode and mode profile as shown in Figure 1(g).

## 3 Topological transport of high-speed data

**Figure 2:**
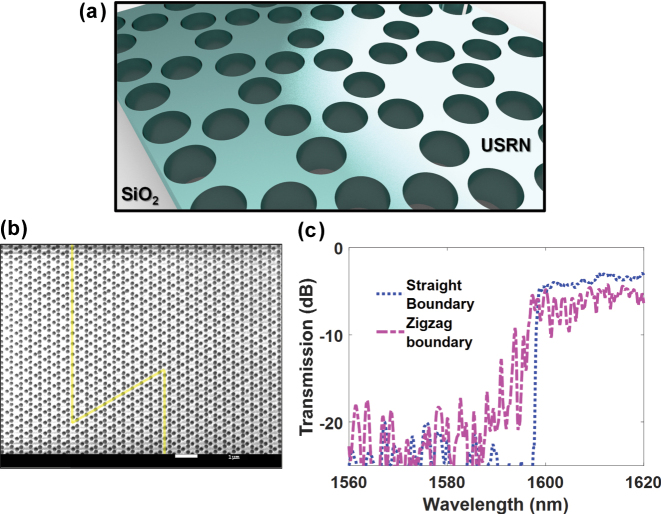
The designs and transmission properties for the topological photonic crystal. (a) Schematic showing the lattice configuration and materials used and (b) scanning electron micrograph of the topological Kagome photonic crystal. The yellow lines show the topological boundary. (c) Transmission spectrum of the topological Kagome photonic crystal with the straight (blue dotted line) and zigzag (fuchsia dashed line) boundary.

Fabrication of the devices is performed on a 300 nm USRN film. The film is deposited using low-temperature chemical vapor deposition onto a 5 μm thermal SiO_2_ on silicon substrate. To define the topological Kagome device, trivial photonic crystal and conventional photonic devices, electron-beam lithography followed by reactive ion etching is performed. The structure is cladded with SiO_2_ deposited using plasma enhanced chemical vapor deposition. Figure 2(a) shows the schematic of the Kagome lattice and material used to implement the device design. We design and fabricate two types of boundaries separating regions with *b* = 0.9*b*_0_ and *b* = 1.1*b*_0_, the first having a straight boundary and the second having a zigzag boundary constituted by four 120° bends. Figure 2(b) shows a scanning electron micrograph of the fabricated topological Kagome device, with the topological boundary marked in yellow. The transmission spectrum of the device with the straight and zigzag boundary is further plotted in Figure 2(c) where it may be observed that the bandgap starts at around 1,598 nm for both devices with similar transmissivity. The topological structure confers robust valley edge transport which makes light propagation within the device immune to backscattering even in the presence of sharp bends.

**Figure 3:**
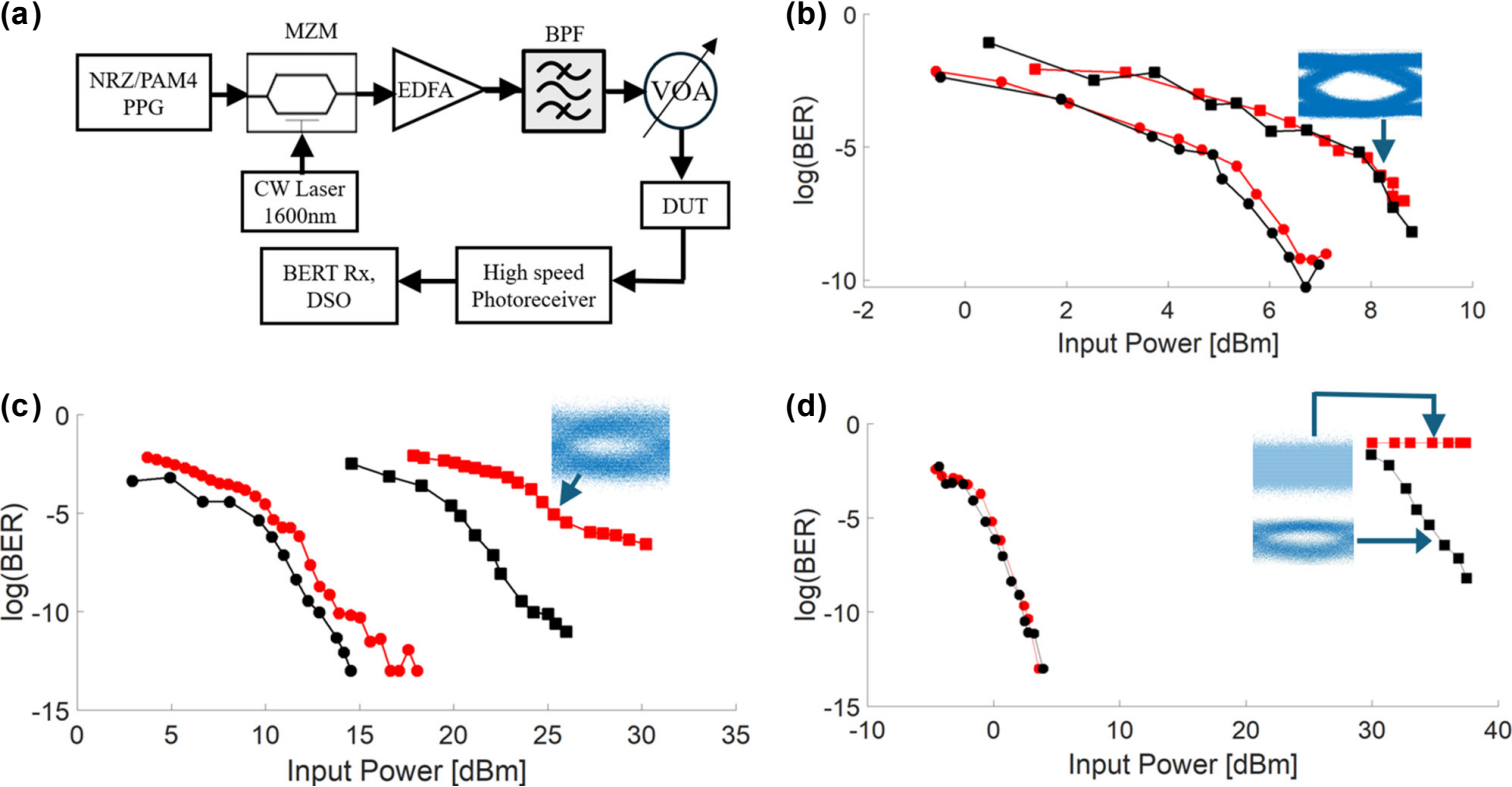
High-speed characterization. (a) Experimental schematic on high-speed transmission. NRZ: non-return-zero. PAM4: pulse amplitude modulation 4-level. PPG: pulse pattern generator. MZM: Mach–Zehnder modulator. CW: continuous wave laser. EDFA: erbium doped fiber amplifier. BPF: bandpass filter. DUT: device under test. VOA: variable optical attenuator. BERT: bit error rate tester. DSO: digital sampling oscilloscope. (b–d) Bit error rate as a function of receiver power of fabricated devices (red). Red square plots are zig zag devices. Red circles denote straight devices. Black plots denote the respective back-to-back transmission. (b) Is topological devices. (c) Trivial devices. (d) Photonic devices.

We performed high speed characterization on the Kagome devices, trivial devices and conventional waveguide. Figure 3(a) shows the experimental setup. A high-speed pulse pattern generator (PPG) generates a 2^31^−1 pseudorandom binary sequence, the signal of which is used to modulate a 1,600.17 nm continuous wave (CW) laser. 1,600.17 nm corresponds to channel 38 of the ITU DWDM L-band 50 GHz grid. This channel is chosen as it is within the passband range of the straight and zigzag devices and within the tail end limit of the preceding L-band erbium doped fiber amplifier (EDFA). The amplified signal was then bandpass-filtered (BPF) to remove amplified spontaneous emission sideband noise before launching into the device under test (DUT). DUT here refers to the Kagome, trivial and conventional waveguides, each with one device with a straight boundary and zigzag boundary. The output of the DUT was demodulated back to an electrical signal using a high-speed photoreceiver, followed by characterizing the bit error rate (BER) at the receiver (Rx) port of the bit error rate tester (BERT) and digital sampling oscilloscope (DSO). In characterizing the BER, the signaling format used at the PPG was 30 Gbps NRZ, which is an intensity modulated direct detection (IMDD) modulation format commonly used for short to mid reach data center communication. In characterizing the BER, the power at the photoreceiver was varied using the variable optical attenuator (VOA) and their corresponding BER recorded and plotted in Figure 3(b)–(d).

Figure 3(b)–(d) show the plot of BER as a function of input power. The input power refers to the power just before entrance to the DUT. The BER is observed to trend downwards logarithmically with increasing receiver power. In this characterization, we compare the BER plots for straight and zigzag devices with respect to their back-to-back counterparts. The back-to-back measurement refers to replacing the DUT with a variable attenuator with an equivalent loss. In Figure 3(b), the power penalty for the topological device with the straight boundary at a BER of 3.8 × 10^−3^ is 0 dB and that for the topological device with the zigzag boundary is 0.2 dB. Here, the power penalty refers to the difference in power required to obtain a desired bit error rate compared to the back-to-back measurement. A BER of 3.8 × 10^−3^ is a commonly used limit in communications systems where hard decision forward error correction (HD.FEC) is employed. Therefore, we adopt this BER value when assessing the power penalty. At this level (equivalently log(BER) = −2.4) it may be observed in Figure 3(b) that a relatively smaller input power of +0.71 dBm was required for the straight boundary, in contrast to a slightly higher power of +3.2 dBm in the zigzag boundary. This represents a marginal increase of 2.4 dB to achieve the same BER.

The eye diagram of the topological zigzag device shown in the inset of Figure 3(b) reveals a clear and open eye. The topological devices yielded a superior power penalty as a result of the device’s ability to withstand backscattering of light. Topological protection in photonic valley Hall insulators manifests through valley-polarized boundary states, which mitigate optical backscattering caused by structural imperfections or defects. The topological boundary state arises from the global Berry phase and is not a localized phase dynamic. A short-range interaction cannot lead to intervalley scattering. Thus, the states allow for one-way propagation, reducing interference and maintaining signal fidelity at high speeds. This is crucial for high-speed signal transmission, as backscattering can lead to signal loss, distortion, and increased BER at high-speed signal.

At the laser wavelength of 1,600.11 nm, there is a 1 dB lower transmission in the zigzag compared to the straight topological device (Figure 3(c)). There is a slightly higher penalty in the high-speed measurements in Figure 3(b). We postulate that this small 1 dB difference could be due to non-idealities in the zigzag propagation. For the zig zag topological device, there could exist interference between forward and a small extent of backward scattered light. Despite some slight non-idealities in the robustness, it may still be observed that the performance at a sharp edge of zig zag topological devices compared to other non-topological photonic devices is significantly better. The slightly different wavelength could have different transmission up to 2 dB level. For different wavelengths, there could be 2∼3 dB degradation in the transmission level. Despite this, the results will still be significantly better in the topological device compared to non-topological devices. The degradation is much bigger for the trivial device (15 dB) and the conventional waveguide (34 dB). In the non-topological zigzag device and the zigzag photonic waveguide, the insertion loss of is similarly high across all wavelengths in our laser’s measurement range from 1,520 nm to 1,620 nm. Therefore, even considering the bandwidth, the topological device is advantageous, providing lower loss transmission in zigzag paths across a wider wavelength range than the other devices. The usable wavelength range for high-speed data transmission in the device shown in Figure 2(c) is from 1,600 nm to 1,620 nm, which would allow 26 100 GHz DWDM channels to reside within. Given the significantly higher loss in the non-topological zigzag and photonic zigzag devices, there is no region within 1,520 nm–1,620 nm where the transmission level is on par with the topological zigzag devices’ stopband region.

Next, we analyze the high-speed characterization for the trivial devices. It may be observed from Figure 3(c) that the power penalty is 1.5 dB for the straight trivial device and 4 dB for zigzag trivial device. At the FEC limit (log(BER) = −2.42 level), the power required was +4.7 dBm for the trivial straight device and significantly larger at +20 dBm for the trivial zigzag devices. This is expected because the insertion loss difference between these 2 devices was around 10 dB. The experimentally measured +20 dBm power requirement is also significantly higher compared to the topological zigzag device (+3.2 dBm).

As the input power is increased, the BER for the trivial zigzag device saturates at around 10^−6^. The nontopological device does not possess topological protection against backscattering. When light propagates through a 120° bend, higher loss is incurred. To overcome higher loss, gain originating from an EDFA is required to impart higher amplification to reach the same BER. However, higher amplification also induces higher amplified spontaneous emission noise which exacerbates the BER. These concurrent effects detrimental to the integrity of the high-speed data causes the BER to plateau at the 10^−6^ level as the receiver power is characterized from −3 dBm to 0 dBm. The inset shows that the eye diagram is visibly closed, though the BER still meets the FEC limits.

Figure 3(d) shows the measurements for conventional photonic waveguides. For the straight devices, the required input power was well below 0 dBm in order to achieve the log(BER) = −2.42 level. For the zig zag devices, the input power was 30 dB higher than this which is a significantly larger power. Despite that, the measured bit error rate for the zigzag photonic devices was still large at 10^−1^ for high receiver powers between +30 dBm and +40 dBm. The absence of topological protection in the photonic devices resulted in large losses from backscattering at the zigzag interfaces, resulting in very high bit error rates. The inset of Figure 3(d) shows the measured eye diagram for the zig zag photonic device, where it is observed that the eye is closed, indicating that the data has undergone significant deterioration. It is observed that the photonic zigzag device did not yield any acceptable BER for all the input powers used. Comparing Figure 3(d) (Photonic zigzag) and Figure 3(b) (Topological zigzag), the latter enjoyed a significant improvement in the BER quantitatively from 10^−1^ to 10^−8^ at the −6 dBm receiver power, all else being equal.

The input power used in the BER measurements for the zigzag topological waveguide ranged from 0 to 9 dBm (Figure 3(b)). This input power is significantly lower than the input power required for the BER measurements in the zigzag photonic waveguides (30–38 dBm; Figure 3(d)). The reason that significantly higher power was needed in the zigzag photonic waveguides is because severe back scattering occurs in the photonic waveguides when the light encounters sharp edges. This significantly impacts the signal integrity and limits integration density. Scattering and backscattering losses occurs at sharp edges in a photonic waveguide. These losses are rooted in the local interaction between the electromagnetic field and the waveguide’s imperfect geometry or material properties. Specifically, sharp corners introduce large reflections and can excite out-of-plane scattering. This process leads to the loss of the guided mode by coupling it to radiation modes. This effect is particularly exacerbated when the bending radius is comparable to the wavelength of light and is also influenced by the distribution of the electric field within the photonic crystal waveguide. For the topological device, the boundary mode is robust because the topological mode is generated on the boundary between two different bulk geometry by long range phenomena, Berry phase. This topological protection leads to minimal loss when light propagates through the sharp bends, thus enabling low input power for the BER measurements shown in Figure 3(b).

**Figure 4:**
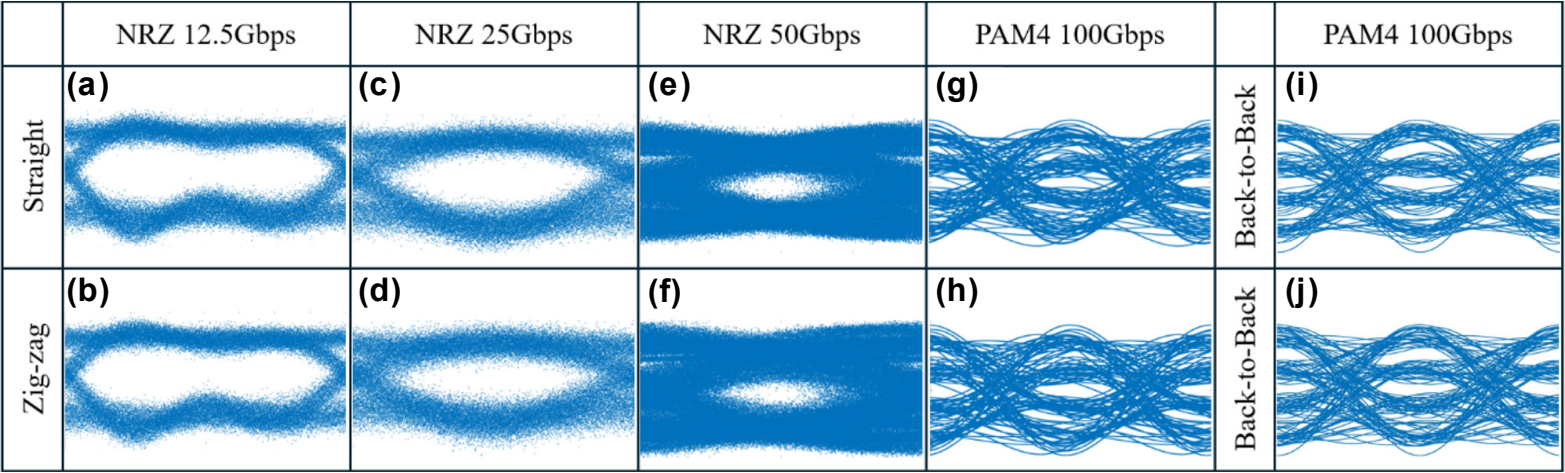
Eye diagram for topological devices (a) NRZ 12.5 Gbps for straight device, (b) NRZ 12.5 Gbps for zig-zag device, (c) NRZ 25 Gbps for straight device, (d) NRZ 25 Gbps for zig-zag device, (e) NRZ 50 Gbps for straight device, (f) NRZ 50 Gbps for zigzag device, (g) PAM-4 100 Gbps for straight device, (h) PAM-4 100 Gbps for zig-zag device. Eye diagram for back-to-back at PAM4 100 Gbps for (i) straight device, (j) zig-zag device.

As industry continues to push for IMDD data transmission rates, such as the 100G single lambda by Cisco Optics, we perform further characterization on the topological devices using 50 Gbps NRZ and 100 Gbps PAM4 at a transmission wavelength of 1,600 nm into the topological straight and zigzag devices. Embedding data bits in the amplitude levels associated with PAM4 and PAM8 modulation formats is one approach to augmenting the data capacity in IMDD transmission. The measured eye diagrams are shown in Figure 4. Here, the digital sampling oscilloscope (DSO) would only output an eye diagram as long as bit error rate is smaller than 10^−3^. This means that the modulated signal was able to achieve sync lock at the DSO end. The measured eye jitter and width of the eye opening are 2.50 ps, 5.75 ps, respectively, for the straight topological devices and similar values of 2.68 ps, 6.61 ps, respectively, for zig zag topological devices. The PAM4 eye diagrams for the straight and zigzag devices are shown in Figure 4(g) and (h) and its back-to-back comparison in Figure 4(i) and (j). This characterization further corroborates the effectiveness of the topological device’s immunity to backscattering for high-speed data transmission even at more advanced modulation formats and higher speeds.

We note that the BER plot versus power for both the straight and zig zag topological devices have a trend that matches closely that of the back-to-back data. This indicates that the high-speed signal experiences negligible signal attenuation without distortion of the high-speed data signal or any additional nonlinear loss induced by high power of signal. We postulate that in the topological device, high-speed signals would have smaller linear loss and minimal distortions even when subjected to extreme operating conditions. The observed decrease in the BER as the signal power is increased is a result of a monotonous error function with the same fixed noise spectral density for light propagation in both the straight and zig zag topological devices. Conversely, the trivial device and the conventional photonic waveguide has different noise spectral density for light propagation in the straight and zigzag configurations. The zigzag propagation has a larger noise spectral density than the straight propagation for the trivial samples, leading to a significantly higher bit error rate at the same input power for the zigzag devices.

Table 1 lists quantum valley Hall devices which have been used for high-speed data transmission. Recently in 2025, Funayama et al. [38] used frequency shift keying (in contrast to IMDD) to demonstrate digital signal transmission in their valley-hall effect based coupler. They demonstrated a BER of 10^−2^ at a frequency deviation of 45 kHz. Valley Hall based power splitters have also been demonstrated at telecommunications wavelengths [*b*]. In this work, Wang et al. demonstrate splitting of 128 Gbps PAM4 data with a 33:67 splitting ratio, yielding a bit error rate of 10–3. Transmission of high-speed data in valley Hall devices has also been reported at terahertz wavelengths, demonstrating their applicability to a broad range of frequencies and associated applications. In this work, bit error rates of 10^−12^ were achieved at data modulation rates of 11 Gbps although the bit error rate was further reported to increase to 10^−2^ when the data rate increased to 13.5 Gbps. In our work, we achieved demonstrated bit error rates of 10^−8^ for 50 Gbps NRZ data. Figure 4 further plots the eye diagrams for 12.5 Gbps, 25 Gbps, 50 Gbps NRZ, and 100 Gbps PAM4. We note further that NRZ and PAM4 modulation are commonly used modulation formats in data center communications, notably being used in transceivers standardized by the CWDM4 [39] and PSM4 [40] multi-source agreements.

**Table 1:** List of quantum valley Hall devices which have been demonstrated for high-speed data transmission, including the modulation formats, rates and bit error rates achieved.

Structure	Modulation format and wavelength	Measured bit error rate	Reference
Terahertz valley-Hall photonic crystal	On-off keying at 0.335 THz	10^−12^ at 11 Gbps and 10^−3^ at 13 Gbps, and 10^−2^ at 13.5 Gbps	Yang et al. [41]
Valley Hall power splitter	PAM4 at 1,550 nm	10^−3^ at 128 Gbps	Wang et al. [42]
Quantum valley Hall coupler	Frequency shift keying (FSK), carrier signal at 360 kHz	10^−2^ at a frequency deviation Δ*f* = 45 kHz.	Funayama et al. [38]
Valley Hall photonic crystal waveguide	NRZ and PAM4 at 1,550 nm	10^−8^ at 50 Gbps	This work

## 4 Discussion

The transmission of high-speed data is one area where topological photonics may impart robustness to. Robustness against artifacts which regular waveguiding methods such as total internal reflection and gap guiding suffer from is one of the most unique attributes of photonic topological insulators. Of note, immunity against disorder in Su–Schreiffer–Heeger waveguides was shown to enable efficient generation of entangled photons in topological photonic systems of different topological invariants, in a Su–Schreiffer–Heeger waveguide [43]. Topological protection can also be effective against not only disorders that preserve the specific chiral symmetry but also disorders that break the chiral symmetry of the system while preserving the underlying topological invariant.

More recently, in photonic anomalous Floquet insulators [44], the topological properties were shown to arise from periodic modulation. This allows for the creation of topological phases that might not exist in static systems. The topological protection in such systems can be inherently tied to the frequency and phase of the driving field. This means that even if the underlying static material has defects or disorder, the dynamic, Floquet-induced topological invariant can still preserve the protected modes. Disorder in the static material properties might be overcome by strong, periodic driving, which effectively circumvents local imperfections. Furthermore, femtosecond laser written topological waveguides were demonstrated to topologically protect the photonic mode at the middle of the photonic bandgap [45], which similar to Ref. [43], is robust against forms of disorder that do not disrupt the chiral symmetry. Most recently, Hu et al. demonstrated topological protection of vortex transport, realized using non-trivial winding in real and momentum space [46]. As a result, a specific orbital angular momentum mode could be selectively retrieved against a background constituted by other excited modes. The range and diversity of optical effects which may benefit from topological protection showcased in these examples underscore opportunities to harness topology for applications and research questions that may be further studied. The previous discussions of disorder usually pertain to Hermitian systems, where energy is conserved. However, many real-world photonic systems are non-Hermitian due to gain, loss, or coupling to an environment. Introducing disorders in these non-Hermitian parameters can lead to complex effects not seen in Hermitian disorder. Recent work has shown that topological invariants can be extended to non-Hermitian systems, like the non-Hermitian skin effect [47], [48], [49] and continued robustness of edge states even in the presence of non-Hermitian disorder. This opens up possibilities for designing robust photonic devices that can operate in lossy environments. For our device, we would like to emphasize that one unique advantage of photonic valley Hall insulators lies in their immunity to disorders that preserve overall valley-contrasting physics. For instance, minor fluctuations in the size or position of individual photonic elements, as long as they do not explicitly mix the valley states, will have little impact on the propagation of light. This is because the robustness stems from a bulk property related to the valley Chern number, which remains invariant even under significant local perturbations. This makes them particularly well-suited for applications where precise fabrication is challenging, offering a pathway to robust waveguiding and mode separation, even in the presence of real-world imperfections.

We have showcased robust valley transport of high-speed data in a topological photonic crystal. 30 Gbps NRZ and 100 Gbps PAM4 data is demonstrated to be efficiently transmitted through compact optical paths comprised of four 120° sharp bends. Compared to reference devices of the same length including a trivial photonic crystal and photonic waveguide, significantly better bit error rates an eye diagrams are achieved for the topological Kagome photonic crystal. Importantly, the bit error rates achieved in the topological device with the zigzag routes are lower than the FEC limit, indicating that the performance exceeds that required for communications systems. This work showcases a promising approach for efficient transmission of high-speed data at telecommunications wavelengths immune to backscattering, potentially enabling significantly smaller photonic integrated circuit footprints to be realized.

In this work, topological protection against backscattering enables high-speed data transmission of NRZ and PAM4 data through zigzag paths with four 120° sharp bends. The robustness against backscattering conferred by the quantum valley Hall effect enables very compact photonic integrated circuits to be implemented. The topological protection is further demonstrated through the significantly better bit error rate and eye diagrams achieved for the zigzag topological device, compared to the zigzag non-topological device and zigzag photonic waveguide. In addition, the device is implemented using ultra-silicon-rich nitride, which is a backend CMOS compatible, low temperature chemical vapor deposition grown material. Therefore, this device can be easily integrated with other CMOS devices and ASICs (application specific integrated circuits). The cost effectiveness and throughput of device manufacturing may be further increased by utilizing wafer-scale processing in the future.

Our work advances the field of topological photonics by demonstrating high-speed data transmission on topological straight and zigzag devices, a critical step toward practical applications. While previous studies have explored topological principles, they have done so with more limited data rates. Specifically, Qi et al. [34] achieved a 25 Gbps signal at 1,550 nm, and while they did reach 100 Gbps, it required wavelength division multiplexing (WDM). In contrast, our study demonstrates a higher single-channel data rate of 50 Gbps NRZ and an ultra-high rate of 100 Gbps using PAM4 modulation on a single channel, pushing the performance limits. Furthermore, unlike the work by Shalaev et al. [50], which focused on backscattering suppression and transmittance, our research validates these topological concepts in a practical communication setting. We also significantly surpass the 11 Gbps data rate reported by Yang et al. [41] at the terahertz wavelength, proving the viability of topological devices for high-speed communication in the near-infrared C-band.

In our work, we study light propagation through zigzag paths in three types of on-chip light guiding structures, including the topological Kagome photonic crystal, the non-topological Kagome photonic crystal, and a photonic waveguide. We note further that high-speed transmission of IMDD data has been reported in other types of on-chip light guiding structures. For example, 56 Gb/s PAM4 data transmission was demonstrated in Su–Schreiffer–Heeger waveguides [51], whereas 50 Gbps PAM4 data transmission was demonstrated over a dielectric waveguide link [52]. These works demonstrated the transmission of high data rates in integrated photonic waveguides albeit slightly lower than in our work. However, unlike the topological waveguides that we report in this paper, these photonic waveguide designs are not robust against backscattering.
